# Heterotrophic Nitrogen Fixation at the Hyper-Eutrophic Qishon River and Estuary System

**DOI:** 10.3389/fmicb.2020.01370

**Published:** 2020-06-24

**Authors:** Eyal Geisler, Anne Bogler, Edo Bar-Zeev, Eyal Rahav

**Affiliations:** ^1^The Zuckerberg Institute for Water Research (ZIWR), The Jacob Blaustein Institutes for Desert Research (BIDR), Ben-Gurion University of the Negev, Beer-Sheva, Israel; ^2^Israel Oceanographic and Limnological Research, National Institute of Oceanography, Haifa, Israel

**Keywords:** N_2_ fixation, polysaccharides, aggregates, estuary, nitrogenase immunolocalization

## Abstract

Planktonic heterotrophic diazotrophs (N_2_-fixers) are widely distributed in marine and freshwater systems, yet limited information is available on their activity, especially in environments with adverse conditions for diazotrophy (e.g., N-rich and oxygenated). Here, we followed the localization and activity of heterotrophic diazotrophs in the hyper-eutrophic N-rich Qishon River—an environment previously considered to be unfavorable for diazotrophy. Our results indicate high heterotrophic N_2_ fixation rates (up to 6.9 nmol N L^–1^ d^–1^), which were approximately three fold higher at an upstream location (freshwater) compared to an estuary (brackish) site. Further, active heterotrophic diazotrophs were capture associated with free-floating aggregates by a newly developed immunolocalization approach. These findings provide new insights on the activity of heterotrophic diazotrophs on aggregates in environments previously considered with adverse conditions for diazotrophy. Moreover, these new insights may be applicable to other aquatic regimes worldwide with similar N-rich/oxygenated conditions that should potentially inhibit N_2_ fixation.

## Introduction

Dissolved inorganic nitrogen (DIN) often controls primary and bacterial production in aquatic systems (Gruber and Galloway, 2008; Moore et al., 2013; Rahav et al., 2018b). While atmospheric dinitrogen (N_2_) is highly abundant, it is unavailable for most microorganisms, except N_2_-fixers that can reduce the N_2_ molecule to ammonium using the nitrogenase enzyme complex (Zehr et al., 2000; Gruber and Galloway, 2008; Sohm et al., 2011). N_2_-fixers, also known as diazotrophs, are a specific group of prokaryotic microorganisms. They are routinely found in a wide range of marine and freshwater environments (Sohm et al., 2011; Zehr, 2011; Luo et al., 2012) and include both autotrophic (Capone et al., 2005; Bergman et al., 2013) and heterotrophic (Riemann et al., 2010; Rahav et al., 2013; Bombar et al., 2016) prokaryotic representatives.

It is generally accepted that N_2_ fixation occurs by cyanobacterial diazotrophs in N-poor, sunlit environments, and that high levels of DIN can inhibit this process (Karl et al., 2002; Gruber, 2008; Knapp, 2012). However, several studies reported unexpectedly high N_2_-fixation rates in N-rich environments such as aphotic waters (Hamersley et al., 2011; Rahav et al., 2013; Benavides et al., 2015), estuaries (Subramaniam et al., 2008; Bentzon-Tilia et al., 2014; Bombar et al., 2016; Pedersen et al., 2018), nutrient-rich coastal waters (Mulholland et al., 2019), and in laboratory settings using N-amended cultures of diazotrophic cyanobacteria (Fu and Bell, 2003; Knapp, 2012). In many of these environments, a high diversity of heterotrophic diazotrophs, rather than phototropic cyanobacterial N_2_-fixers, is often found (Riemann et al., 2010), suggesting other controls and possibly mechanisms that enable N_2_ fixation by these heterotrophic microorganisms. While numerous studies have investigated the limiting factors for cyanobacterial diazotrophs (reviewed in Moore et al., 2013), much less is known about the nutrient controls of aquatic heterotrophic diazotrophs. One of the ways heterotrophic diazotrophs could flourish in the abovementioned aquatic environments is by adopting a particle-associated lifestyle rather than being free-living (Rahav et al., 2013; Bombar et al., 2016; Farnelid et al., 2019).

To date, several studies suggested that free-floating aggregates may be favorable loci for heterotrophic N_2_ fixation due to its unique physicochemical characteristics (Paerl and Carlton, 1988; Rahav et al., 2013, 2016; Pedersen et al., 2018 and see more details below). Free-floating aggregates often referred to as marine, lake, or river snow (Grossart et al., 1997; Simon et al., 2002; Lundgreen et al., 2019) are ubiquitous throughout the aquatic environment and play a central role in marine and freshwater food webs as micro-islands with intense microbial activity (Azam and Malfatti, 2007; Bar-Zeev and Rahav, 2015; Arnosti et al., 2016). In comparison to the surrounding waters, these aggregates usually contain high levels of organic and inorganic compounds, trace elements, as well as detrital matter and fecal pellets held together by a sticky scaffold comprised of proteins and polysaccharides such as transparent exopolymer particles (TEP) (Ploug and Grossart, 2000; Passow, 2002). These aggregates usually have a high C:N ratio compared to the typical ∼6.6:1 Redfield ratio, thereby inducing N-limiting conditions for different microbes, including heterotrophic diazotrophs. Indeed, once formed, aggregates are often heavily colonized by various microorganisms (del Giorgio and Cole, 1998; Simon et al., 2002; Bar-Zeev et al., 2012), including heterotrophic diazotrophic representatives affiliated with *nifH* Cluster III (e.g., *Desulfovibrio putealis*) and Cluster 1 (e.g., *Azoarcus* sp., *Dechloromonas aromatic*, and *Rhodobacterales*) (Pedersen et al., 2018; Farnelid et al., 2019). Bacterial abundances associated with aggregates are several orders of magnitudes higher compared to the surrounding water (Grossart and Simon, 1993; Turley and Mackie, 1994; Kiørboe et al., 2002). Bacteria/archaea associated with aggregates solubilize and re-mineralize organic matter at higher rates compared to free-living bacteria (Grossart and Simon, 1998), resulting in “hotspots” of intense microbial activity (Long and Azam, 2001; Bar-Zeev and Rahav, 2015; Arnosti et al., 2016), potentially also to heterotrophic diazotrophs (Rahav et al., 2013; Bombar et al., 2016; Farnelid et al., 2019). Further, the high aerobic respiration by bacteria colonizing aggregates (including heterotrophic diazotrophs), combined with slow diffusion rates, may lead to reduced oxygen levels toward the aggregate’s center of ≤80% air saturation and occasionally even to anoxia conditions (Paerl and Prufert, 1987; Klawonn et al., 2015). Such oxygen-reduced micro-zones may greatly benefit diazotrophs since the nitrogenase enzyme may be irreversibly damaged by O_2_ (Gruber, 2008). Moreover, O_2_-protective mechanisms used by different diazotrophs have been shown to consume much of the energy required for N_2_ fixation (Großkopf and LaRoche, 2012). Currently, it is unknown how the nitrogenase in heterotrophic diazotrophs is protected from O_2_, and it is possible that aggregates/fecal pellets provide low O_2_ micro-environment that “enable” N_2_ fixation. There is currently limited information on the activity of planktonic heterotrophic diazotrophs and their association with aggregates in eutrophic freshwater environments.

In this study, we measured heterotrophic N_2_ fixation in a DIN-rich aquatic environment and established a direct association between active heterotrophic diazotrophs (that synthesized nitrogenase) and polysaccharide aggregates. To this end, we sampled the eutrophic Qishon River (SE Mediterranean Sea) at two sites with different C:N and N:P ratios that may affect heterotrophic diazotrophy. Specifically, heterotrophic N_2_ fixation, bacterial abundance, bacterial activity, and TEP concentrations were measured along with a suite of different physicochemical variables during summer and winter. Since the focus of this study was to examine heterotrophic N_2_ fixation, water collected was incubated in the dark and with the addition of a photosynthetic inhibitor. Further, we used an immunolabeling approach to specifically localize diazotrophs actively expressing nitrogenase on aggregates comprising polysaccharides. Our results suggest that particles could be loci for N_2_ fixation by heterotrophic bacteria in eutrophic environments traditionally considered to be unfavorable for diazotrophy.

## Materials and Methods

### Study Sites and Sampling Strategy

**FIGURE 1:**
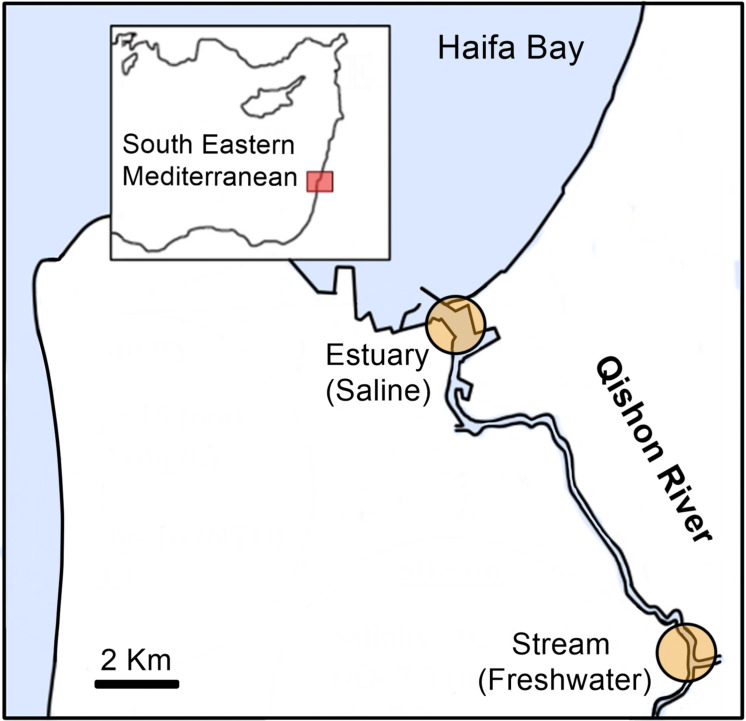
Map of the estuary and stream sampling sites in the Qishon River. Estuary water was sampled twice in the summer (August 2014, September 2017) and twice in winter (November 2013, January 2018), whereas the stream was sampled once in the winter (January 2018) and once in the summer (September 2017). Additional information on the water phytochemical characteristics is provided in Table 1.

Surface water (∼0.3 m) was collected from the Qishon River at two sites; one located downstream at the entrance of the estuary to the coast (hereafter referred to as “estuary,” Lat: 32°48′44.42”N, Lon. 35°02′00.6′′E), and one upstream (hereafter referred to as “stream,” Lat: 32°43′34.5′′N, Lon. 35°05′53.2′′E) (Figure 1). The estuary station was sampled in November 2013 (late summer/autumn), August 2014, September 2017 (summer), and January 2018 (winter). Stream water was sampled in September 2017 (summer) and January 2018 (winter) (Supplementary Table S1). The collected water was divided into four pre-cleaned (10% HCl and autoclaved) 1-L Nalgene bottles and amended with artificial estuary water enriched in ultra-pure ^15^N_2_ (Mohr et al., 2010). Specific focus on the activity of heterotrophic diazotrophs (rather than cyanobacterial diazotrophs) was achieved by incubating the samples for 48 h in the dark with 3-(3,4-dichlorophenyl)-1,1-dimethylurea (DCMU, 50 μM final concentration, Sigma-Aldrich D2425) to impair phototrophic activity (Clavier and Boucher, 1992). An additional bottle was collected and filtered immediately to measure the natural abundance of dissolved ^15^N_2_ (no tracer addition) (Montoya et al., 1996). Previous studies showed that prolonged dark incubation (48 h) and addition of DCMU can halt autotrophic diazotrophy, thereby providing valuable information specifically on heterotrophic N_2_ fixation (Rahav et al., 2015, 2016; Benavides et al., 2018).

**TABLE 1:** Summary of the physicochemical characteristics of the Qishon River during the summer (September 2017) and winter (January 2018) samplings.

Location	Season	Salinity (ppt)	DO (mg L^–^^1^)	pH	Turbidity (NTU)	TOC (mg L^–^^1^)	TN (mg L^–^^1^)	TP (mg L^–^^1^)	NO_2_+ NO_3_ (mg L^–^^1^)	PO_4_(mg L^–^^1^)
Stream	Summer	0.5	7.5	7.6	67	38	7.8	0.2	6.04	0.14
	Winter	0.3	7.0	7.8	44	141	21.0	0.7	10.35	0.27
Estuary	Summer	21	6.2	8.1	3	18	3.7	0.3	1.13	0.06
	Winter	11	6.5	8.0	17	72	5.8	0.4	3.08	0.18

In addition, the association between aggregates and diazotrophs was visualized using a recently developed confocal microscopy approach at the conclusion of the incubations (under dark+DCMU conditions as well as under ambient light as controls). Natural aggregates collected from the stream and estuary are shown in Geisler et al. (2019).

### Physicochemical Characteristics

Light penetration through the water column was measured using a Secchi disk (Holmes, 1970). Salinity was measured using an electrical conductivity meter (EC-30 cond, Phoenix Instruments), dissolved oxygen (DO) using an oxygen meter (ProODO, YSI), pH using a pH meter (Cybercan pH 11, Eutech), and turbidity using a turbidity meter (Tu-2016, Lutron). All sensors were calibrated based on the manufacturer’s instructions. Total phosphorus (TP), orthophosphate (PO_4_), and nitrate+nitrite (NO_3_+NO_2_) were measured using a segmented flow Technicon Auto-Analyzer II (AA-II) system. The detection limit for P was 2.5 × 10^–4^ mg L^–1^, while for N it was 5.6 × 10^–4^ mg L^–1^ (Sisma-Ventura and Rahav, 2019). Total organic carbon (TOC) and total nitrogen (TN) were analyzed with a TOC analyzer (Multi N/C, Analytik-Jena, Germany, detection limit; 0.3 mg L^–1^) and calibrated using a five-point calibration procedure (Kowalski et al., 2009). These physicochemical measurements were carried out to characterize the initial environmental characteristics of the water at both sites and were measured during September 2017 and January 2018 (same as the visualization approach described below).

### Transparent Exopolymer Particles (TEP) Concentration

Subsamples (<100 mL) were filtered through a 0.4-μm polycarbonate filter (GE Water & Process Technologies) using low pressure (<150 mbar) to avoid breakdown of the aggregates at the end of the incubation (T_48_). Filters were stained with a 0.2% Alcian blue solution and washed three times with deionized water. TEP was extracted using sulfuric acid (80%) for 2 h. The supernatant (1 ml) absorbance was measured using a spectrophotometer at 787 nm wavelength (Thermo GENESYTM). Alcian-blue dye was calibrated against known concentrations of the purified polysaccharide gum-xanthan (GX) (Passow and Alldredge, 1995).

### Bacterial Abundance (BA)

Water samples (1.7 mL) were fixed with glutaraldehyde (final concentration 0.02%, Sigma-Aldrich G7651) at T_48_, flash frozen in liquid nitrogen, and stored at −80°C until analyses within a few days. Prior to analyses, the samples were thawed at room temperature, sonicated for 30 s (Qsonica, Q55 Sonicator), and EDTA (2.5 μM) was added to release the cells associated/attached to the aggregates to the water media and stained with 0.5 nM SYBR Green for 10 min in the dark. Subsamples were analyzed with an Attune NEXT flow cytometer (Applied Biosystems) using a blue laser signal. Samples were run at a constant flow rate of 25 μL min^–1^ and cells were differentiated using green fluorescence and side scatter (Vaulot and Marie, 1999). Cell size range in an Attune NEXT system is ∼0.5–70 μm. Beads (0.93 μm, Polysciences) were run in parallel as a size standard. Blank samples of stained sterile river water (0.2 μm) and deionized water were also run and their reads were removed from the total bacterial counts.

### Bacterial Production (BP)

Triplicate samples (1.7 mL) were spiked at T_48_ with 2 nM of ^3^H-leucine (123 Ci mmol^–1^, Perkin Elmer) and incubated for 3–4 h under ambient temperature in the dark. Incubations were terminated by adding 100% trichloroacetic acid, followed by applying the micro-centrifugation technique (Simon and Azam, 1989; Smith et al., 1992). Disintegration per minute (DPM) was measured using a Tri-CARB 4810 TR (Packard) liquid scintillation counter. Incorporation of leucine was converted to carbon by a conservative factor of 3.1 kg C mol^–1^ with an isotope dilution factor of 2.0.

### Heterotrophic Dinitrogen (N_2_) Fixation

Measurements were taken by adopting the ^15^N_2_-enriched water method (Mohr et al., 2010), without degassing pre-step. The enriched medium was prepared before each sampling event (summer or winter) by injecting ^15^N_2_ gas (99%, Cambridge Isotopes, lot # NLM-363-PK) into pre-filtered (0.2 μm) artificial estuary water at a 1:100 (*v:v*) ratio (i.e., 1 cc of ^15^N_2_ gas per 100 ml of artificial water). The enriched stock was vigorously shaken to completely dissolve the ^15^N_2_ gas bubble. The same ^15^N_2_ tank was used to prepare the enriched water for both the summer and winter sampling campaigns. We used the atom % value provided previously using similar methodology and recipe for preparing the enriched seawater (Wilson et al., 2012) as no membrane-introduction mass spectrometry (MIMS) measurements were available. The percent enrichment can vary between batches when preparing ^15^N_2_-enriched water. However, since we used the same ^15^N_2_-enriched water in each sampling event, a comparison between the estuary and stream sites at the Qishon River reflects the measured differences between the sites.^15^N_2_ stock was added to the 1 L experimental bottles (5% of total sample volume, Rahav et al., 2015) and incubated for 48 h in the dark with DCMU to impair phototrophic diazotrophy (Rahav et al., 2015; Benavides et al., 2018) and focus specifically on heterotrophic diazotrophs. Preliminary experiment from the Northern Red Sea (and not the Qishon River) showed that N_2_ fixation rates were insignificantly different following dark alone or dark+DCMU incubations, thereby refuting any concern that the addition of DCMU may result in dissolved organic matter supply derived from autotrophs death/lysing which may favors heterotrophic microbial activity (including diazotrophy) (Supplementary Figures S1, S2). The samples were filtered through pre-combusted (450°C, 4.5 h) glass fiber filters and dried overnight at 60°C. A minimum of 10 μg particulate N (PN) per filter ensured adequate sample mass to resolve small differences in N isotope ratios (White et al., 2020). The samples were analyzed on a CE Instruments NC2500 elemental analyzer interfaced to a Thermo-Finnigan Delta Plus XP isotope ratio mass spectrometer (IRMS). Heterotrophic N_2_ fixation was calculated according to Mulholland et al. (2006) using N solubility factors described by Weiss (1970). One bottle without ^15^N enrichment was used for natural abundance of N_2_ at each station. Standard curves were processed with each sample run to determine N for isotope ratio mass spectrometry. Samples were run only when standard curves had *R*^2^ > 0.99. At masses >4.7 μg N, precision for the atom percent ^15^N measurement was ±0.0001% based on daily calibrations made in association with sample runs and calibrations averaged over runs made over several years. Based on natural abundance, N mass on the filters, incubation times, and the precision of mass spectrometer, the detection limit for ^15^N uptake was ∼0.02 nmol N L^–1^ d^–1^. This detection limit was lower by ∼70–95% of the heterotrophic N_2_ fixation rates measured in this study, thus providing credibility to the results.

### Visualization of Active Diazotrophs on Polysaccharide-Based Aggregates

The staining protocol is described in detail in Geisler et al. (2019). Briefly, sub-samples (∼20 mL) were collected from the microcosm bottles at the end of incubations (T_48_) during the September 2017 and January 2018 sampling campaigns (Supplementary Table S1), gently filtered (>150 mbar) on a 0.4 μm filter and stained for the following: (i) diazotrophs were tagged with an anti-nitrogenase (Agrisera Antibodies AS01 021A) solution (6 μg mL^–1^) followed by an anti-chicken antigen conjugated to a FITC fluorophore (Thermo Fisher Scientific A-11039) for 45 min, (ii) Total bacteria were stained with DAPI for 45 min, (iii) The polysaccharide matrix of the aggregates was stained with the fluorescent lectin concanavalin A (ConA, 200 μg mL^–1^, Ex. 630 nm, Em. 647 nm, Thermo Fisher Scientific C11252) for 40 min, and (iv) Heterotrophic bacteria were distinguished from phototrophic cyanobacteria by subtracting the auto-fluorescence of phycoerythrin (Ex. 490 nm and Em. 580 nm). Although indicative to most cyanobacteria (not necessarily diazotrophs), phycoerythrin is not found in all strains, and thus, it is possible that some phototrophic diazotrophs were not identified. The stained samples were imaged with a Zeiss confocal laser scanning microscope (CLSM 510 Meta) equipped with 405 nm diode, 488 nm Aragon, and 633 nm helium-neon lasers. Captured CLSM images were processed using ZEN (blue edition). We surmised that most of the bacteria that have translated the nitrogenase enzyme would actively fix N_2_, resulting in a positive FITC fluorophore staining (Geisler et al., 2019). Additional controls for the visualization approach are provided in Supplementary Figures S2–S4 and include (i) testing if the addition of DCMU introduced carbon-rich substrates stimulated heterotrophic N_2_ fixation (Supplementary Figure S2); (ii) incubations under ambient light that capture the activity of both autotrophic and heterotrophic diazotrophic activity (Supplementary Figure S3); and (iii) testing unspecific staining of the secondary antibody (Supplementary Figure S4).

### Statistical Analyses

The statistical significance between the values of heterotrophic N_2_ fixation, bacterial production (BP), bacterial abundance (BA) and TEP at the “estuary” or “stream” stations was determined using a student’s *t*-test using XLSTAT 2016 (Microsoft, New York, United States) with a confidence level of 95% (α = 0.05).

## Results and Discussion

The Qishon River is a eutrophic system with water outflow into the Haifa Bay, SE Mediterranean Sea (Kress and Herut, 1998). Water in the Qishon River flows through several industrial facilities located upstream (e.g., fertilizer plants, an oil refinery, and a sewage treatment plant) that discharge high amounts of nutrients (105 ton N y^–1^ and 2.5 ton P y^–1^, N:P = 42:1)^1^, usually resulting in a steep eutrophic to oligotrophic gradient (Eliani-Russak et al., 2013; Vachtman et al., 2013; Bar-Zeev and Rahav, 2015). During both seasons, the stream water exhibited low salinity (0.3–0.5 ppt) and high turbidity (44–67 Nephelometric Turbidity Units, NTU), whereas the estuary water was saltier (11–21 ppt) and less turbid (3–17 NTU) (Table 1). Light penetration was limited to <30 cm, and the water was oxygenated (>6 mg L^–1^) in all samplings and locations (Table 1 and Figure 1). Total organic carbon (TOC, 18–141 mg L^–1^), total nitrogen (TN, 3.7–21.0 mg L^–1^), and total phosphorus (TP, 0.2–0.4 mg L^–1^) measurements were high and considered as hyper-eutrophic based on the NOAA reference values for rivers and estuaries (National Oceanic and Atmospheric Administration (NOAA), 1996). Similarly, DIN (NO_2_+NO_3_, 1.13–10.35 mg L^–1^) and orthophosphate (PO_4_, 0.06–0.27 mg L^–1^) were high, resulting in DIN:PO_4_ of 17–40:1 (Table 1). Previous studies reported that DIN concentrations higher than 1 μM (≈0.01 mg L^–1^), C:N ratio lower than ∼6.6:1, and DIN:PO_4_ >16:1 should significantly impair cyanobacterial N_2_ fixation rates (Gruber and Galloway, 2008; Knapp, 2012) and likely also heterotrophic diazotrophy (Rahav et al., 2016; Geisler et al., 2019).

**FIGURE 2:**
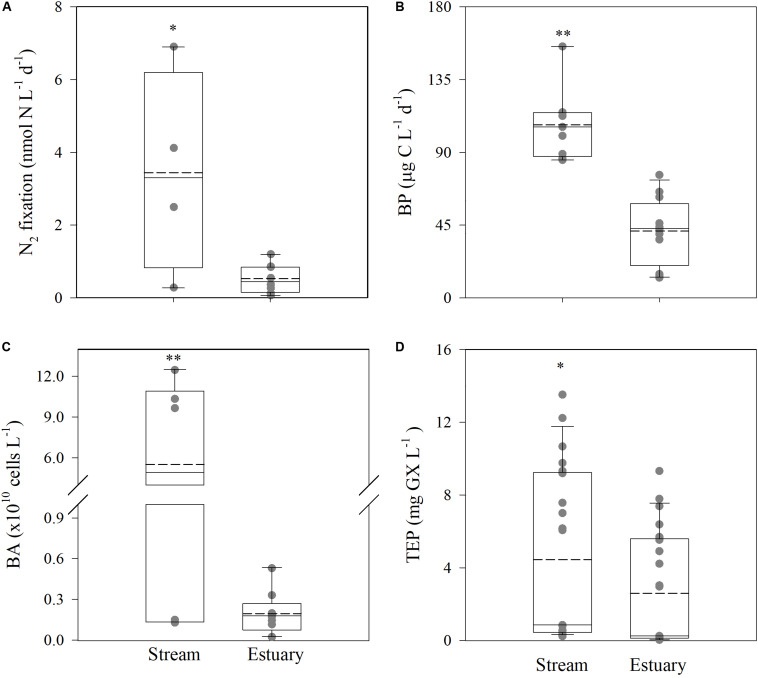
Heterotrophic N_2_-fixation rates **(A)**, BP rates **(B)**, BA **(C)**, and TEP concentrations **(D)** in the Qishon stream and estuary systems. The data shown were compiled from the summer and the winter sampling campaigns (Supplementary Table S1). Whiskers indicate the interquartile range (25th to 75th percentile) of the dataset. The mean values are shown as a solid line. Asterisks indicate the statistical significant differences between the Qishon stream and the estuary stations (*t*-test) where *P < 0.05; **P < 0.01. The averaged value in each sampling campaign and location is shown in Supplementary Table S2, and the actual measured values used to generate the box-plots are shown in gray.

Despite the unfavorable chemical conditions that prevailed along the river, heterotrophic N_2_ fixation rates (up to 6.9 nmol N L^–1^ d^–1^, Figure 2A) were several folds higher than the typical values reported in the nearby oligotrophic southeastern Mediterranean Sea (usually <0.05 nmol N L^–1^ d^–1^, Raveh et al., 2015; Rahav et al., 2016, 2018a; Rahav and Bar-Zeev, 2017). Corresponding heterotrophic N_2_ fixation rates were seven fold higher at the hyper-eutrophic stream (median ∼3.30 nmol N L^–1^ d^–1^) compared to the estuary (median ∼0.45 nmol N L^–1^ d^–1^) (*t*-test, *P* = 0.01, Figure 2A). Nonetheless, these rates were comparable to studies from other eutrophic estuaries and fjords (range values reported ∼2 to ∼80 nmol N L^–1^ d^–1^) (Subramaniam et al., 2008; Bentzon-Tilia et al., 2014; Pedersen et al., 2018), suggesting that such environments should be included in calculations of addition of N through N_2_ fixation in future global aquatic N balance. Concurrently, BP (12.5–155.5 μg C L^–1^ d^–1^), BA (0.03–12.5 × 10^10^ cells L^–1^), and TEP (0.04–13.5 mg xanthan-gum L^–1^) were also higher at the stream compared to the estuary sites by two to three fold (Figures 2B–D).

**FIGURE 3:**
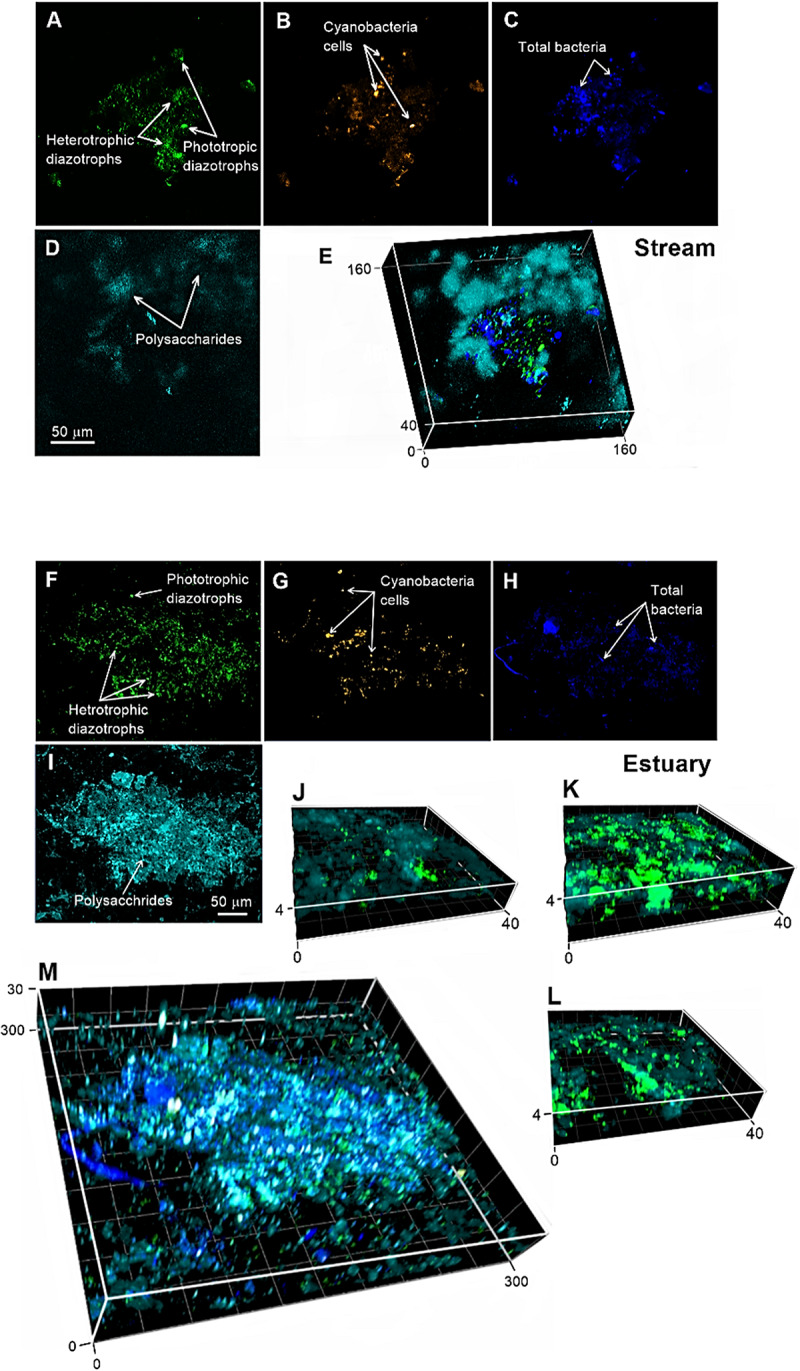
Visualization of the natural microbial population in the stream **(A–E)** and estuary **(F–M)** captured by a confocal laser scanning microscope during September 2017 and January 2018 (Supplementary Table S1) at T_48_. **(A,F)** active diazotrophs tagged by immunolabeling (green); **(B,G)** cyanobacteria phycoerythrin autofluorescence (orange); **(C,H)**, total bacteria stained with DAPI (dark blue); and **(D,I)** polysaccharides stained with ConA (light blue). **(E,M)** The 3D images show the superimposed signals of the different stains. **(J–L)** 3D images show the zoom in of the aggregates in different locations. The axes of the superimposed images are reported in micrometers. For additional magnified confocal images see Supplementary Figure S5.

The identification of active diazotrophs in association with aggregates comprising polysaccharides such as TEP in the Qishon River was visualized using a recently developed immunolabeling approach (Geisler et al., 2019). This approach enabled direct visualization of active diazotrophs that synthesized the nitrogenase enzyme on aggregates comprising a polysaccharides matrix, along with cyanobacteria and other (not necessarily diazotrophs) prokaryotic/eukaryotic microorganisms (Figure 3 and Supplementary Figure S2). Using this direct visualization approach, we demonstrated that polysaccharide-based aggregates collected from the Qishon River (estuary and stream) were colonized by dense communities of active heterotrophic diazotrophs (Figure 3 and Supplementary Figure S5). Additional microscopic analyses taken after 48 h incubation at ambient light conditions clearly show that cyanobacteria colonized most of the aggregates area but only few were also diazotrophs (Supplementary Figure S3). Additionally, incubation for 48 h under dark+DCMU conditions of the same water indicated that only few unicellular cyanobacteria have synthesized the nitrogenase enzyme (i.e., were “active”). We cannot rule out that some of the colonizing phototrophic (cyanobacteria) diazotrophs were mixotrophs, namely bacteria that can “switch” between heterotrophic metabolism to carbon fixation via photosynthesis, rather than obligatory phototrophs. Recent studies demonstrated that the cyanobacterium *Trichodesmium*, previously characterized as phototrophs, may, in fact, use mixotrophic metabolism (Benavides et al., 2017, 2020). Similarly, the unicellular cyanobacterial diazotroph *Cyanothece* have been shown to take up carbohydrates and amino acids (Feng et al., 2010). Thus, it is possible that under dark+DCMU conditions, mixotrophic diazotrophs could also be captured, hence the phycoerythrin signal on our aggregates.

Our immunolocalization images from the Qishon River (Figure 3 and Supplementary Figure S5) suggest that these microenvironments are active hubs for heterotrophic diazotrophs. These images therefore support previous reports that correlated between aggregates such as TEP and heterotrophic N_2_ fixation (Rahav et al., 2013, 2016 and abovementioned references). It also supports reports of 16S rRNA and *nifH* gene amplicon sequencing showing that particle samples are colonized by N_2_-fixing microbes of both autotrophic (e.g., *Trichodesmium*, *Crocosphaera, Nodularia spumigena*, symbionts of diatoms, and UCYN-A) and heterotrophic (e.g., *Desulfovibrio putealis*, *γ24774A11*, *Azoarcus* sp., *Rhodobacterales*, and *Dechloromonas aromatic*) representatives (Klawonn et al., 2015; Pedersen et al., 2018; Farnelid et al., 2019).

Colonization of aggregates by diazotrophs, especially in N-rich ecosystems, may provide microenvironments favoring heterotrophic N_2_ fixation due to the following reasons: (i) The polysaccharide matrix of the aggregate can be degraded by the inhabiting microbes, thus providing additional energy sources to heterotrophic diazotrophs in the form of labile carbon (Bar-Zeev and Rahav, 2015), (ii) Aggregates comprising a polysaccharides matrix such as TEP would often exhibit high C:N ratio (∼20:1) compared to the Redfield ratio (∼6.6:1) in the surrounding water (Passow, 2002; Engel et al., 2004), thereby “inducing” N-limiting conditions that favor diazotrophs (Gruber, 2008), (iii) The aggregate matrix may provide microenvironments with reduced O_2_ concentration as it is respired toward the center of the aggregate (Paerl and Prufert, 1987; Klawonn et al., 2015; Bombar et al., 2016), thus minimizing oxidative damages to the nitrogenase enzyme (Milligan et al., 2007). This may reduce the cellular energetic cost tunneled to segregate the nitrogenase from O_2_ in oxygenated environments (Großkopf and LaRoche, 2012), and (iv) Aggregates often comprise high levels of trace metals such as Fe and Mo that are adsorbed to the particles from the environment (Vicente et al., 2009) and are essential to the nitrogenase function (Berman-Frank et al., 2001).

## Conclusion

The chemical conditions that prevailed at the eutrophic Qishon River should potentially inhibit heterotrophic N_2_ fixation, especially given the high concentrations of DIN and the high N:P ratio. Nonetheless, we measured high rates of heterotrophic N_2_ fixation, which were several folds higher compared to rates reported from many oligotrophic marine environments, including the neighboring eastern Mediterranean Sea. Generally, the highest heterotrophic N_2_ fixation rates were measured in the Qishon stream, where lower C:N and higher N:P ratios were measured compared to the estuary sampling site. These results are in agreement with the few studies that report on unexpectedly high diazotrophic activity in N-rich aquatic environments. We also show evidence of dense colonies of active heterotrophic diazotrophs associated with polysaccharides-based aggregates in N-rich environments. We suggest that heterotrophic diazotrophs are supported by aggregates and may gain different biochemical advantages compared to planktonic diazotrophs in environments with unfavorable conditions for diazotrophy such as the eutrophic Qishon River. It is likely that the close physical proximity of diazotrophs (as well as other bacteria) to each other on aggregates (including TEP) may enhance the hydrolysis efficiency of carbohydrates and uptake of low-molecular weight organic compounds. In turn, energy gained through this C-rich hydrolysis can be tunneled to nitrogenase synthesis and heterotrophic N_2_ fixation. We stress that future studies should use methods that couple visualization of individual cells and heterotrophic N_2_-fixation rates supported by aggregates such as TEP (e.g., immunolocalization and NanoSIMS). These studies should also investigate the diversity, activity, and biomass of particle-associated diazotrophs as a function of the environmental and particle’s characteristics. Finally, the contribution of cyanobacterial and heterotrophic diazotrophy by aggregate associated cells to the bulk N_2_ fixation should be quantified via dedicated studies in different marine and freshwater environments. These will enable a better understanding of the role of aggregate-associated diazotrophy throughout the marine and freshwater environments, which can later be implemented in N budget studies and models.

## Data Availability Statement

All datasets generated for this study are included in the article/Supplementary Material.

## Author Contributions

EG, ER, and EB-Z conceived and designed the experiments, performed the samplings, and wrote the manuscript. EG, AB, ER, and EB-Z analyzed the data. ER and EB-Z contributed reagents, materials, and analysis tools. All authors contributed to the article and approved the submitted version.

## Conflict of Interest

The authors declare that the research was conducted in the absence of any commercial or financial relationships that could be construed as a potential conflict of interest.

## Footnotes

1 www.kishon.org.il

## References

[B1] Arnosti C. Ziervogel K. Yang T. Teske A. (2016). Oil-derived marine aggregates - hot spots of polysaccharide degradation by specialized bacterial communities. *Deep. Res. Part II Top. Stud. Oceanogr.* 129 179–186. 10.1016/j.dsr2.2014.12.008

[B2] Azam F. Malfatti F. (2007). Microbial structuring of marine ecosystems. *Nat. Rev. Microbiol.* 5 782–791. 10.1038/nrmicro1747 17853906

[B3] Bar-Zeev E. Berman-Frank I. Girshevitz O. Berman T. (2012). Revised paradigm of aquatic biofilm formation facilitated by microgel transparent exopolymer particles. *Proc. Natl. Acad. Sci. U.S.A.* 109 9119–9124. 10.1073/pnas.1203708109 22615362 PMC3384133

[B4] Bar-Zeev E. Rahav E. (2015). Microbial metabolism of transparent exopolymer particles during the summer months along a eutrophic estuary system. *Front. Microbiol.* 6:403. 10.3389/fmicb.2015.00403 26042092 PMC4436900

[B5] Benavides M. Berthelot H. Duhamel S. Raimbault P. Bonnet S. (2017). Dissolved organic matter uptake by Trichodesmium in the Southwest Pacific. *Sci. Rep.* 7:41315. 10.1038/srep41315 28117432 PMC5259775

[B6] Benavides M. Duhamel S. Van Wambeke F. Shoemaker K. M. Moisander P. H. Bonnet S. (2020). Dissolved organic matter stimulates N2 fixation and nifH gene expression in Trichodesmium. *FEMS Microbiol. Lett.* 367:fnaa034 10.1017/CBO9781107415324.004 32083662

[B7] Benavides M. Moisander P. H. Berthelot H. Dittmar T. Grosso O. Bonnet S. (2015). Mesopelagic N2 fixation related to organic matter composition in the Solomon and Bismarck seas (southwest pacific). *PLoS One* 10:e143775. 10.1371/journal.pone.0143775 26659074 PMC4684240

[B8] Benavides M. Martias C. Elifantz H. Berman-Frank I. Dupouy C. Bonnet S. (2018). Dissolved organic matter influences N2 fixation in the New Caledonian lagoon (Western Tropical South Pacific). *Front. Mar. Sci.* 5:89 10.3389/fmars.2018.00089

[B9] Bentzon-Tilia M. Traving S. J. Mantikci M. Knudsen-Leerbeck H. Hansen J. L. Markager S. (2014). Significant N2 fixation by heterotrophs, photoheterotrophs and heterocystous cyanobacteria in two temperate estuaries. *ISME J.* 9 1–13. 10.1038/ismej.2014.119 25026373 PMC4303622

[B10] Bergman B. Sandh G. Lin S. Larsson J. Carpenter E. J. (2013). Trichodesmium - A widespread marine cyanobacterium with unusual nitrogen fixation properties. *FEMS Microbiol. Rev.* 37 286–302. 10.1111/j.1574-6976.2012.00352.x 22928644 PMC3655545

[B11] Berman-Frank I. Cullen J. T. Shaked Y. Sherrell R. M. Falkowski P. G. (2001). Iron availability, cellular iron quotas, and nitrogen fixation in Trichodesmium. *Limnol. Oceanogr.* 46 1249–1260. 10.4319/lo.2001.46.6.1249

[B12] Bombar D. Paerl R. W. Riemann L. (2016). Marine non-cyanobacterial diazotrophs: moving beyond molecular detection. *Trends Microbiol.* 24 916–927. 10.1016/j.tim.2016.07.002 27476748

[B13] Capone D. G. Burns J. A. Montoya J. P. Subramaniam A. Mahaffey C. Gunderson T. (2005). Nitrogen fixation by Trichodesmium spp.: an important source of new nitrogen to the tropical and subtropical North Atlantic Ocean. *Glob. Biogeochem. Cycles* 19 1–17. 10.1029/2004GB002331

[B14] Clavier C. G. J. Boucher G. (1992). The use of photosynthesis inhibitor (DCMU) for in situ metabolic and primary production studies on soft bottom benthos. *Hydrobiologia* 246 141–145. 10.1007/BF00014701

[B15] del Giorgio P. A. Cole J. J. (1998). Bacterial growth efficiency in natural aquatic systems. *Annu. Rev. Ecol. Syst.* 29 503–541. 10.1146/annurev.ecolsys.29.1.503

[B16] Eliani-Russak E. Herut B. Sivan O. (2013). The role of highly sratified nutrient-rich small estuaries as a source of dissolved inorganic nitrogen to coastal seawater, the Qishon (SE Mediterranean) case. *Mar. Pollut. Bull.* 71 250–258. 10.1016/j.marpolbul.2013.02.001 23485104

[B17] Engel A. Thoms S. Riebesell U. Rochelle-Newall E. Zondervan I. (2004). Polysaccharide aggregation as a potential sink of marine dissolved organic carbon. *Nature* 428 929–932. 10.1038/nature02453 15118723

[B18] Farnelid H. Turk-Kubo K. Ploug H. Ossolinski J. E. Collins J. R. Van Mooy B. A. S. (2019). Diverse diazotrophs are present on sinking particles in the North Pacific Subtropical Gyre. *ISME J.* 13 170–182. 10.1038/s41396-018-0259-x 30116043 PMC6299005

[B19] Feng X. Bandyopadhyay A. Berla B. Page L. Wu B. Pakrasi H. B. (2010). Mixotrophic and photoheterotrophic metabolism in Cyanothece sp. ATCC 51142 under continuous light. *Microbiology* 156 2566–2574. 10.1099/mic.0.038232-0 20430816

[B20] Fu F. X. Bell P. R. F. (2003). Factors affecting N2 fixation by the cyanobacterium Trichodesmium sp. GBRTRLI101. *FEMS Microbiol. Ecol.* 45 203–209. 10.1016/S0168-6496(03)00157-0 19719631

[B21] Geisler E. Bogler A. Rahav E. Bar-zeev E. (2019). Direct detection of heterotrophic diazotrophs associated with planktonic aggregates. *Sci. Rep.* 9 1–9. 10.1038/s41598-019-45505-4 31243322 PMC6594930

[B22] Grossart H. -P. Simon M. (1993). Limnetic macroscopic organic aggregates (lake snow): occurrence, characteristics, and microbial dynamics in Lake Constance. *Limnol. Oceanogr.* 38 532–546. 10.4319/lo.1993.38.3.0532

[B23] Grossart H. -P. Simon M. Logan B. E. (1997). Formation of macroscopic organic aggregates (lake snow) in a large lake: the significance of transparent exopolymer particles, plankton, and zooplankton. *Limnol. Oceanogr.* 42 1651–1659. 10.4319/lo.1997.42.8.1651

[B24] Grossart H. P. Simon M. (1998). Significance of limnetic organic aggregates (lake snow) for the sinking flux of particulate organic matter in a large lake. *Aquat. Microb. Ecol.* 15 115–125. 10.3354/ame015115

[B25] Großkopf T. LaRoche J. (2012). Direct and indirect costs of dinitrogen fixation in Crocosphaera watsonii WH8501 and possible implications for the nitrogen cycle. *Front. Microbiol.* 3:236 10.3389/fmicb.2012.00236 22833737 PMC3401090

[B26] Gruber N. (2008). “The marine nitrogen cycle: overview and challenges,” in Nitrogen in the Marine Environment. *Chapter* 1 1–50. 10.1016/B978-0-12-372522-6.00001-3

[B27] Gruber N. Galloway J. N. (2008). An Earth-system perspective of the global nitrogen cycle. *Nature* 451 293–296. 10.1038/nature06592 18202647

[B28] Hamersley M. Turk K. Leinweber A. Gruber N. Zehr J. Gunderson T. (2011). Nitrogen fixation within the water column associated with two hypoxic basins in the Southern California Bight. *Aquat. Microb. Ecol.* 63 193–205. 10.3354/ame01494

[B29] Holmes R. W. (1970). The secchi disk in turbid coastal waters. *Limnol. Oceanogr.* 15 688–694. 10.4319/lo.1970.15.5.0688

[B30] Karl D. Michaels A. Bergman B. Capone D. (2002). Dinitrogen fixation in the world’s oceans. *Biogeochemistry* 57 47–98. 10.1007/978-94-017-3405-9_2

[B31] Kiørboe T. Grossart H. -P. Ploug H. Tang K. (2002). Mechanisms and rates of colonisation of sinking aggregates. *Appl. Environ. Microbiol.* 68 3996–4006. 10.1128/AEM.68.8.3996 12147501 PMC124032

[B32] Klawonn I. Bonaglia S. Bruchert V. Ploug H. (2015). Aerobic and anaerobic nitrogen transformation processes in N2-fixing cyanobacterial aggregates. *ISME J.* 9 1456–1466. 10.1038/ismej.2014.232 25575306 PMC4438332

[B33] Knapp A. N. (2012). The sensitivity of marine N2 fixation to dissolved inorganic nitrogen. *Front. Microbiol.* 3:374. 10.3389/fmicb.2012.00374 23091472 PMC3476826

[B34] Kowalski N. Dellwig O. Beck M. Grunwald M. Fischer S. Piepho M. (2009). Trace metal dynamics in the water column and pore waters in a temperate tidal system: response to the fate of algae-derived organic matter. *Ocean Dyn.* 59 333–350. 10.1007/s10236-009-0192-7

[B35] Kress N. Herut B. (1998). Hypernutrification in the oligotrophic Eastern Mediterranean: a study in Haifa Bay (Israel). *Estuar. Coast. Shelf Sci.* 46 645–656. 10.1006/ecss.1997.0302

[B36] Long R. A. Azam F. (2001). Antagonistic interactions among marine pelagic bacteria. *Appl. Environ. Microbiol.* 67:4975 10.1128/AEM.67.11.4975 PMC93260 11679315

[B37] Lundgreen R. B. C. Jaspers C. Traving S. J. Ayala D. J. Lombard F. Grossart H. P. (2019). Eukaryotic and cyanobacterial communities associated with marine snow particles in the oligotrophic Sargasso Sea. *Sci. Rep.* 9 1–12. 10.1038/s41598-019-45146-7 31222051 PMC6586830

[B38] Luo Y. -W. Doney S. C. Anderson L. A. Benavides M. Berman-Frank I. Bode A. (2012). Database of diazotrophs in global ocean: abundance, biomass and nitrogen fixation rates. *Earth Syst. Sci. Data* 4 47–73. 10.5194/essd-4-47-2012

[B39] Milligan A. J. Berman-Frank I. Gerchman Y. Dismukes G. C. Falkowski P. G. (2007). Light-dependent oxygen consumption in nitrogen-fixing cyanobacteria plays a key role in nitrogenase protection. *J. Phycol.* 43 845–852. 10.1111/j.1529-8817.2007.00395.x

[B40] Mohr W. Großkopf T. Wallace D. W. R. Laroche J. (2010). Methodological underestimation of oceanic nitrogen fixation rates. *PLoS One* 5:e12583. 10.1371/journal.pone.0012583 20838446 PMC2933240

[B41] Montoya J. P. Voss M. Kaehler P. Capone D. G. (1996). A simple, high precision, high sensitivity tracer assay for dinitrogen fixation. *Appl. Environ. Microbiol.* 62 986–993. 10.1128/aem.62.3.986-993.1996 16535283 PMC1388808

[B42] Moore C. M. Mills M. M. Arrigo K. R. Berman-Frank I. Bopp L. Boyd P. W. (2013). Processes and patterns of oceanic nutrient limitation. *Nat. Geosci.* 6 701–710. 10.1038/ngeo1765

[B43] Mulholland M. R. Bernhardt P. W. Heil C. A. Bronk D. A. Neil J. M. O. (2006). Nitrogen fixation and release of fixed nitrogen by Trichodesmium spp. in the Gulf of Mexico. *Limnol. Oceanogr.* 51 1762–1776. 10.4319/lo.2006.51.4.1762

[B44] Mulholland M. R. Bernhardt P. W. Winder B. N. Selden C. R. Chappell P. D. Clayton S. (2019). High rates of N_2_ fixation in temperate, Western North Atlantic coastal waters expand the realm of marine diazotrophy global biogeochemical cycles. *Glob. Biogeochem. Cycles* 33, 826–840. 10.1029/2018GB006130

[B100] National Oceanic and Atmospheric Administration (NOAA) (1996). *NOAA’s Estuarine Eutrophication Survey. Volume 1: South Atlantic Region*. Silver Spring: Office of Ocean Resources Conservation Assessment, 50.

[B45] Paerl H. W. Carlton R. (1988). Control of nitrogen fixation by oxygen depletion in surface-associated microzones. *Nature* 332 3–5.3347243

[B46] Paerl H. W. Prufert L. E. (1987). Oxygen-poor microzones as potential sites of microbial N2 fixation in nitrogen-depleted aerobic marine waters. *Appl. Environ. Microbiol.* 53 1078–1087. 10.1128/aem.53.5.1078-1087.1987 16347337 PMC203813

[B47] Passow U. (2002). Transparent exopolymer particles (TEP) in aquatic environments. *Prog. Oceanogr.* 55 287–333. 10.1016/s0079-6611(02)00138-6

[B48] Passow U. Alldredge A. L. (1995). A dye-binding assay for the spectrophotometric measurement of transparent exopolymer particles (TEP). *Limonol. Oceanogr.* 40 1326–1335. 10.4319/lo.1995.40.7.1326

[B49] Pedersen J. N. Bombar D. Paerl R. W. Riemann L. (2018). Diazotrophs and N2-fixation associated with particles in coastal estuarine waters. *Front. Microbiol.* 9:2759. 10.3389/fmicb.2018.02759 30505296 PMC6250843

[B50] Ploug H. Grossart H. P. (2000). Bacterial growth and grazing on diatom aggregate: respiratory carbon turnover as a function of aggregate size and sinkig velocity. *Limnol. Oceanogr.* 45 1467–1475. 10.4319/lo.2000.45.7.1467

[B51] Rahav E. Bar-Zeev E. (2017). Sewage outburst triggers Trichodesmium bloom and enhance N2 fixation rates. *Sci. Rep.* 7:4367. 10.1038/s41598-017-04622-8 28663560 PMC5491490

[B52] Rahav E. Bar-Zeev E. Ohayon S. Elifantz H. Belkin N. Herut B. (2013). Dinitrogen fixation in aphotic oxygenated marine environments. *Front. Microbiol.* 4:227. 10.3389/fmicb.2013.00227 23986748 PMC3753716

[B53] Rahav E. Belkin N. Paytan A. Herut B. (2018a). Bacterioplankton response to desert dust deposition in the coastal waters of the southeastern Mediterranean Sea; A four year in-situ survey. *Atmos* 9 1–15. 10.3390/atmos9080305

[B54] Rahav E. Giannetto M. Bar-Zeev E. (2016). Contribution of mono and polysaccharides to heterotrophic N2 fixation at the eastern Mediterranean coastline. *Sci. Rep.* 6:27858. 10.1038/srep27858 27306501 PMC4910064

[B55] Rahav E. Herut B. Mulholland M. Belkin N. Elifantz H. Berman-Frank I. (2015). Heterotrophic and autotrophic contribution to dinitrogen fixation in the Gulf of Aqaba. *Mar. Ecol. Prog. Ser.* 522 67–77. 10.3354/meps11143

[B56] Rahav E. Raveh O. Hazan O. Gordon N. Kress N. Silverman J. (2018b). Impact of nutrient enrichment on productivity of coastal water along the SE Mediterranean shore of Israel - A bioassay approach. *Mar. Pollut. Bull.* 127 559–567. 10.1016/j.marpolbul.2017.12.048 29475698

[B57] Raveh O. David N. Rilov G. Rahav E. (2015). The temporal dynamics of coastal phytoplankton and bacterioplankton in the eastern mediterranean sea. *PLoS One* 10:e140690 10.1371/journal.pone.0140690 26474399 PMC4608699

[B58] Riemann L. Farnelid H. Steward G. (2010). Nitrogenase genes in non-cyanobacterial plankton: prevalence, diversity and regulation in marine waters. *Aquat. Microb. Ecol.* 61 235–247. 10.3354/ame01431

[B59] Simon M. Azam F. (1989). Protein content and protein synthesis rates of planktonic marine bacteria. *Mar. Ecol. Prog. Ser.* 51 201–213. 10.3354/meps051201

[B60] Simon M. Grossart H. P. Schweitzer B. Ploug H. (2002). Microbial ecology of organic aggregates in aquatic ecosystems. *Aquat. Microb. Ecol.* 28 175–211. 10.3354/ame028175

[B61] Sisma-Ventura G. Rahav E. (2019). DOP stimulates heterotrophic bacterial production in the oligotrophic Southeastern Mediterranean coastal waters. *Front. Microbiol.* 10:1913. 10.3389/fmicb.2019.01913 31474972 PMC6706821

[B62] Smith D. C. Smith D. C. Azam F. Azam F. (1992). A simple, economical method for measuring bacterial protein synthesis rates in seawater using 3H-leucine. *Mar. Microb. Food Web* 6 107–114.

[B63] Sohm J. Webb E. Capone D. (2011). Emerging patterns of marine nitrogen fixation. *Nat. Rev. Microbiol.* 9 499–508. 10.1038/nrmicro2594 21677685

[B64] Subramaniam A. Yager P. L. Carpenter E. J. Mahaffey C. Bjokman K. Cooley S. SKustka A. B. (2008). Amazon River enhances diazotrophy and carbon sequestration in the tropical North Atlantic Ocean. *Proc. Natl. Acad. Sci. U.S.A.* 105 10460–10465. 10.1029/2006GB002751 18647838 PMC2480616

[B65] Turley C. M. Mackie P. J. (1994). Biogeochemical significance of attached and free-living bacteria and the flux of particles in the NE Atlantic Ocean. *Mar. Ecol. Prog. Ser.* 115 191–204. 10.3354/meps115191

[B66] Vachtman D. Sandler A. Greenbaum N. Herut B. (2013). Dynamics of suspended sediment delivery to the Eastern Mediterranean continental shelf. *Hydrol. Process.* 27 1105–1116. 10.1002/hyp.9265

[B67] Vaulot D. Marie D. (1999). Diel variability of photosynthetic picoplankton in the equatorial Pacific. *JGR Ocean* 104 3297–3310. 10.1029/98JC01333

[B68] Vicente I. Ortega-Retuerta E. Romera O. Morales-Baquero R. Reche I. (2009). Contribution of transparent exopolymer particles to carbon sinking flux in an oligotrophic reservoir. *Biogeochemistry* 96 13–23. 10.1007/s10533-009-9342-8

[B69] Weiss R. F. (1970). The solubility of nitrogen, oxygen and argon in water and seawater. *Deep. Res. Oceanogr. Abstr.* 17 721–735. 10.1016/0011-7471(70)90037-9

[B70] White A. E. Granger J. Selden C. Gradoville M. R. Potts L. Bourbonnais A. (2020). A critical review of the 15N2 tracer method to measure diazotrophic production in pelagic ecosystems. *Limnol. Oceanogr. Methods* 18 129–147. 10.1002/lom3.10353

[B71] Wilson S. T. Böttjer D. Church M. J. Karl D. M. (2012). Comparative assessment of nitrogen fixation methodologies, conducted in the oligotrophic north pacific ocean. *Appl. Environ. Microbiol.* 78 6516–6523. 10.1128/AEM.01146-12 22773638 PMC3426697

[B72] Zehr J. P. (2011). Nitrogen fixation by marine cyanobacteria. *Trends Microbiol.* 19 162–173. 10.1016/j.tim.2010.12.004 21227699

[B73] Zehr J. P. Carpenter E. J. Villareal T. A. (2000). New perspectives on nitrogen-fixing microorganisms in tropical and subtropical oceans. *Trends Microbiol.* 8 68–73. 10.1016/s0966-842x(99)01670-4 10664599

