# Supplementary material for: Heterotrophic Nitrogen Fixation at the Hyper-Eutrophic Qishon River and Estuary System
Source: Front Microbiol. 2020 Jun 24;11:1370. doi: 10.3389/fmicb.2020.01370 (PMC7326945; doi:10.3389/fmicb.2020.01370)
Supplement: Supplementary file 1 [file Data_Sheet_1.docx]

**Supporting Information**

**Table S1** – Summary of all sampling events and the total number of samples for each variable tested.

| **Parameter** | **Location** | **Sampling events** | | | | **Total measurements (#)** | **Data presentation** |
| --- | --- | --- | --- | --- | --- | --- | --- |
|  |  | Nov. 13 | Aug. 14 | Sept. 17 | Jan. 18 |  |  |
| Salinity, DO, turbidity, pH | Estuary | --- | --- | √ | √ | 2 | Table 1 |
|  | Stream | --- | --- | √ | √ | 2 |  |
| Organic & inorganic nutrients | Estuary | --- | --- | √ | √ | 2 | Table 1 |
|  | Stream | --- | --- | √ | √ | 2 |  |
| N_2_ fixation | Estuary | √ | √ | √ | √ | 4* | Figure 2 |
|  | Stream | --- | --- | √ | √ | 2** |  |
| BA | Estuary | √ | √ | √ | √ | 4* | Figure 2 |
|  | Stream | --- | --- | √ | √ | 2** |  |
| BP | Estuary | √ | √ | √ | √ | 4* | Figure 2 |
|  | Stream | --- | --- | √ | √ | 2** |  |
| TEP | Estuary | √ | √ | √ | √ | 4* | Figure 2 |
|  | Stream | --- | --- | √ | √ | 2** |  |
| Confocal microscopy | Estuary | --- | --- | √ | √ | All aggregates in 20 ml | Figure 3 |
|  | Stream | --- | --- | √ | √ | All aggregates in 20 ml |  |

* In biological triplicates, namely 12 measurements in total.

** In biological triplicates, namely 6 measurements in total.

**Table S2** – Summary of the measured biological variables measured in the different locations along the Qishon River. Data shown are the averages and their corresponding standard deviation. The compiled data is shown in Figure 2 of the main manuscript. N.A – not available.

| **Sampling location** | **Sampling date** | **N_2_ fixation  (nmol N L^-1^ d^-1^)** | **BP  (µg C L^-1^ d^-1^)** | **BA  (x10^10^ cells L^-1^)** | **TEP  (mg GX L^-1^)** |
| --- | --- | --- | --- | --- | --- |
| Stream | Sept. 2017 | 4.4±5.4 | 21±8 | 1.0±0.1 | 0.3±0.1 |
|  | January 2018 | 3.2±3.4 | 107±22 | 13.6±4.4 | 9.2±2.5 |
|  |  |  |  |  |  |
| Estuary | Nov. 2013 | 0.61±0.12 | 62±10 | 0.2±0.1 | 3.0±0.5 |
|  | Aug. 2014 | 0.53±0.10 | 26±8 | 0.1±0.1 | 1.1±0.4 |
|  | Sept. 2017 | 0.26±0.07 | N.A | 0.3±0.1 | 0.5±0.2 |
|  | January 2018 | 0.84±0.01 | 50±14 | 0.1±0.0 | 5.7±2.0 |

**
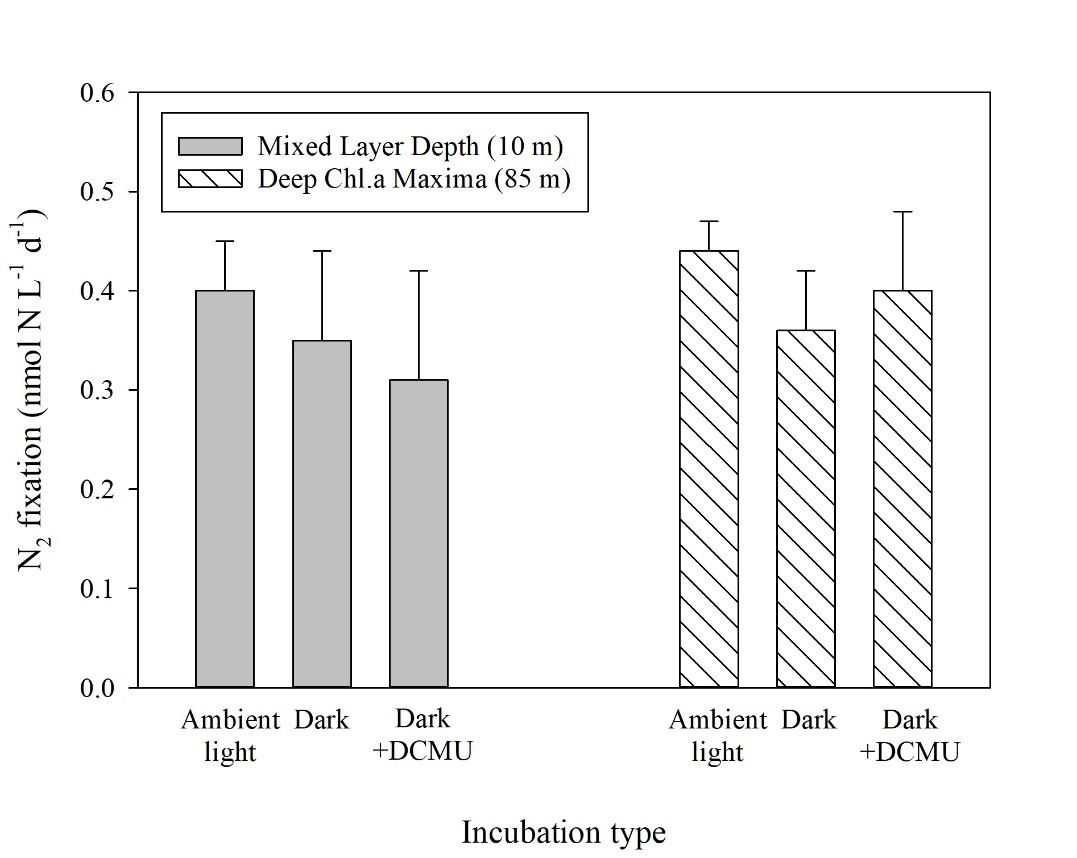
**

**Figure S1 –**N_2_ fixation rates measured in the mixed layer depth (10 m, gray) and the DCM (85 m, stripped white) following incubations under ambient light, dark, and dark+DCMU for 24 h. Seawater samples were collected from the Northern Red Sea in July 2012 (for more details see Rahav et al., (2015).


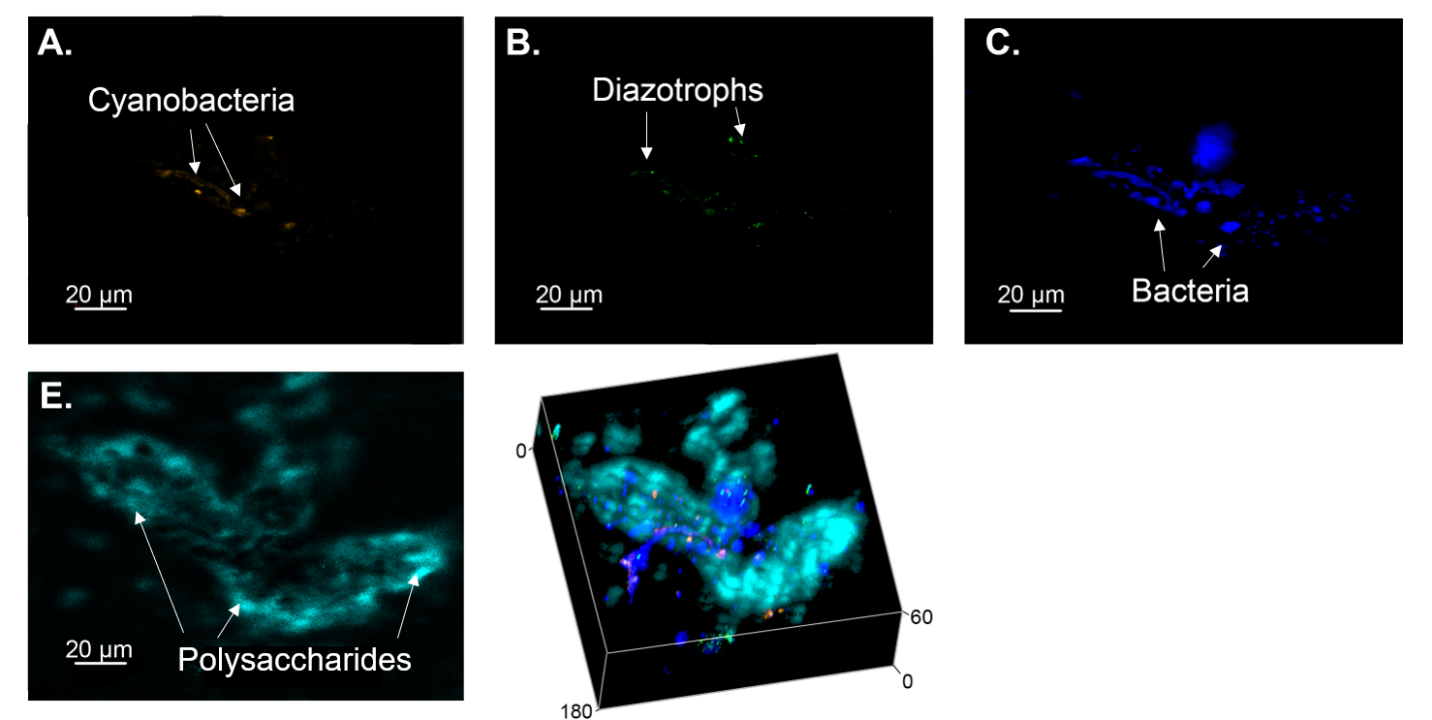


**Figure S2** - Visualization of the natural microbial population in the stream captured by a confocal laser scanning microscope following dark incubation for 48 h without DCMU. (A) cyanobacteria phycoerythrin auto-fluorescence; (B) active diazotrophs tagged by immunolabeling; (C), total bacteria stained with DAPI; and (D) polysaccharides stained with ConA. 3D image shows the superimposed signals of the different stains. The axis of the superimposed images show size in µm.


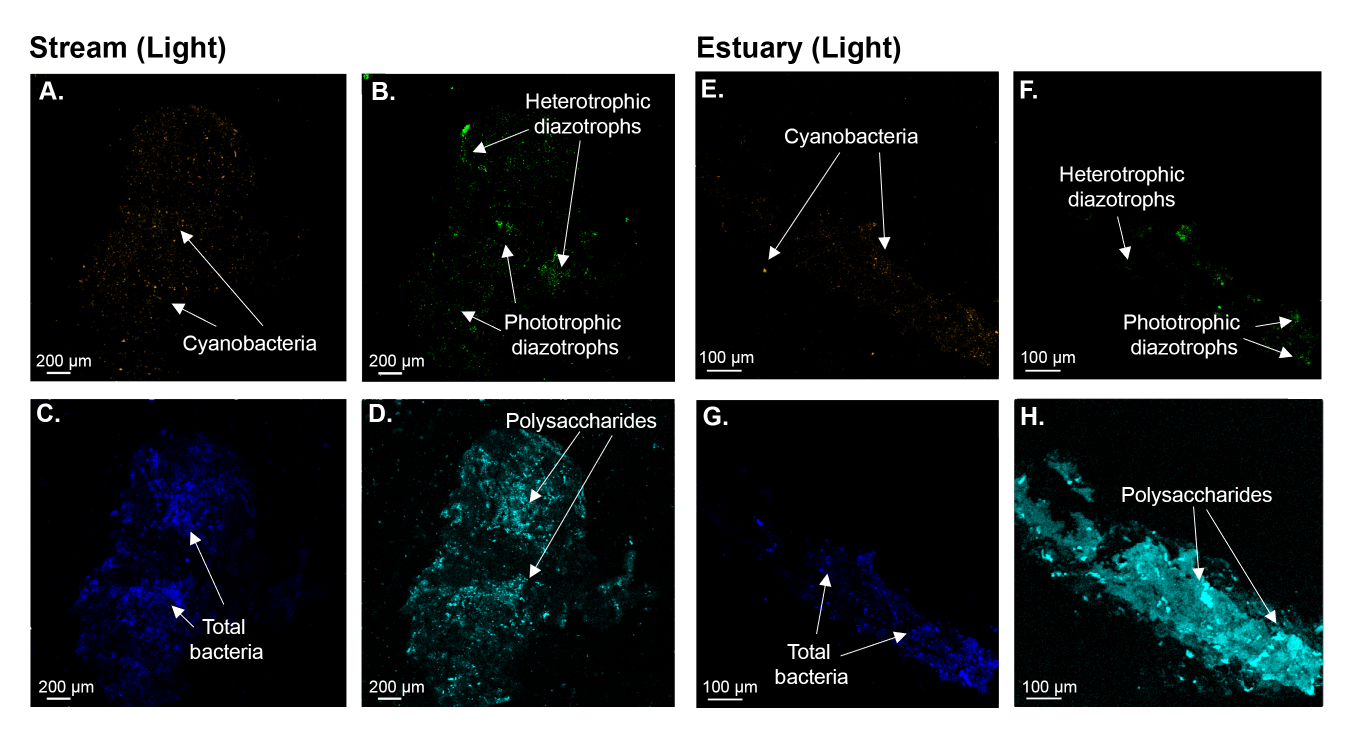


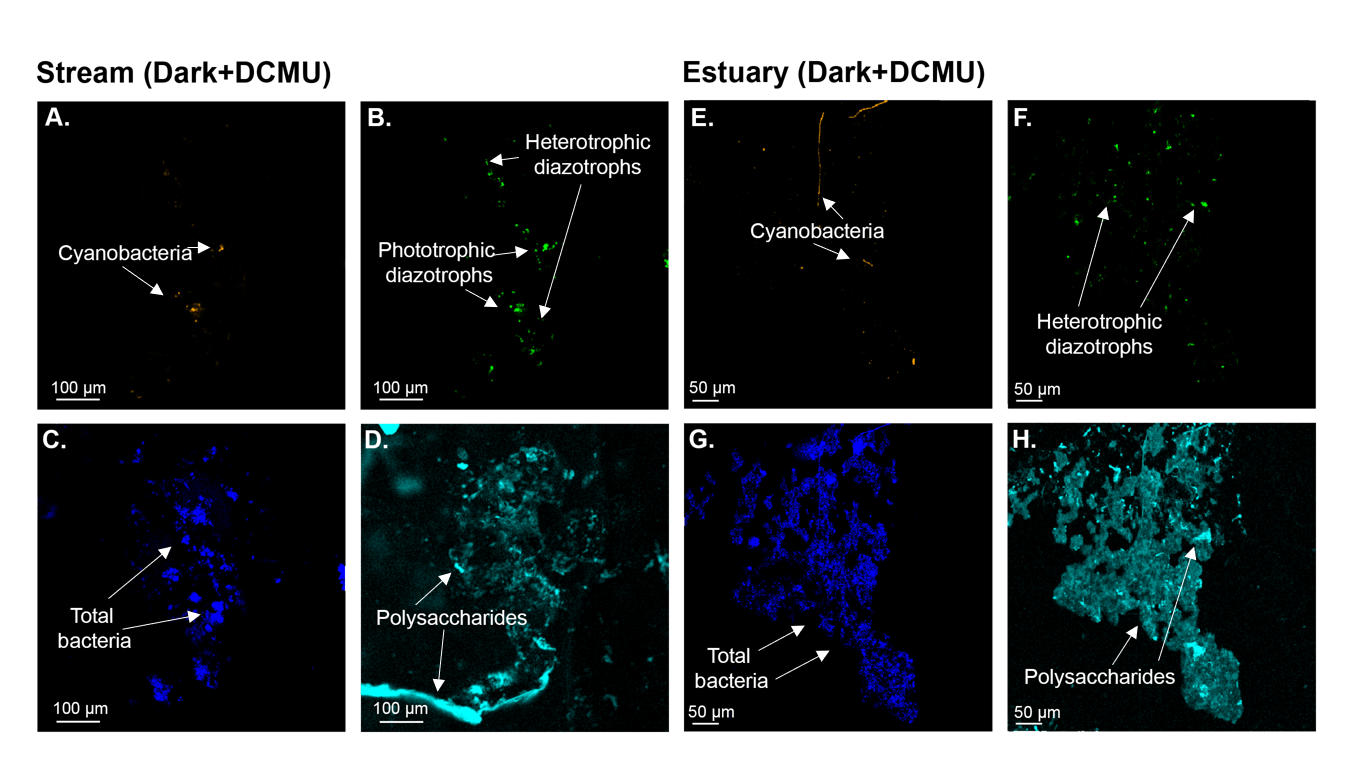
**Figure S3** – **Upper panel**: Visualization of the natural microbial population in the stream (A-D) and estuary (E-H) captured by a confocal laser scanning microscope following ambient light incubation for 48 h. (A, E) cyanobacteria phycoerythrin autofluorescence; (B, F) active diazotrophs tagged by immunolabeling; (C, G), total bacteria stained with DAPI; and (D, H) polysaccharides stained with ConA. **Lower panel:** Visualization of the natural microbial population in the stream (A-D) and estuary (E-H) captured by a confocal laser scanning microscope following dark incubation for 48 h and DCMU addition to impair photosynthesis. (A, E) cyanobacteria phycoerythrin autofluorescence; (B, F) active diazotrophs tagged by immunolabeling; (C, G), total bacteria stained with DAPI; and (D, H) polysaccharides stained with ConA.


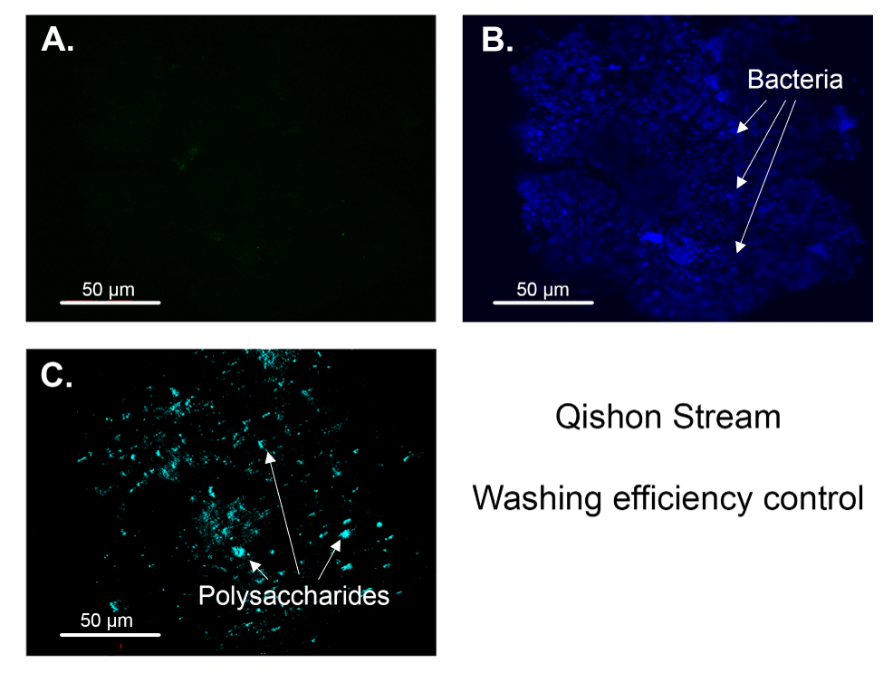


**Figure S4 -** Visualization of washing efficiency of the natural microbial population in the stream captured by a confocal laser scanning microscope following dark+DCMU incubation for 48 h. (A) green fluorophore signal (secondary antibody); (B), total bacteria stained with DAPI; and (C) polysaccharides stained with ConA.


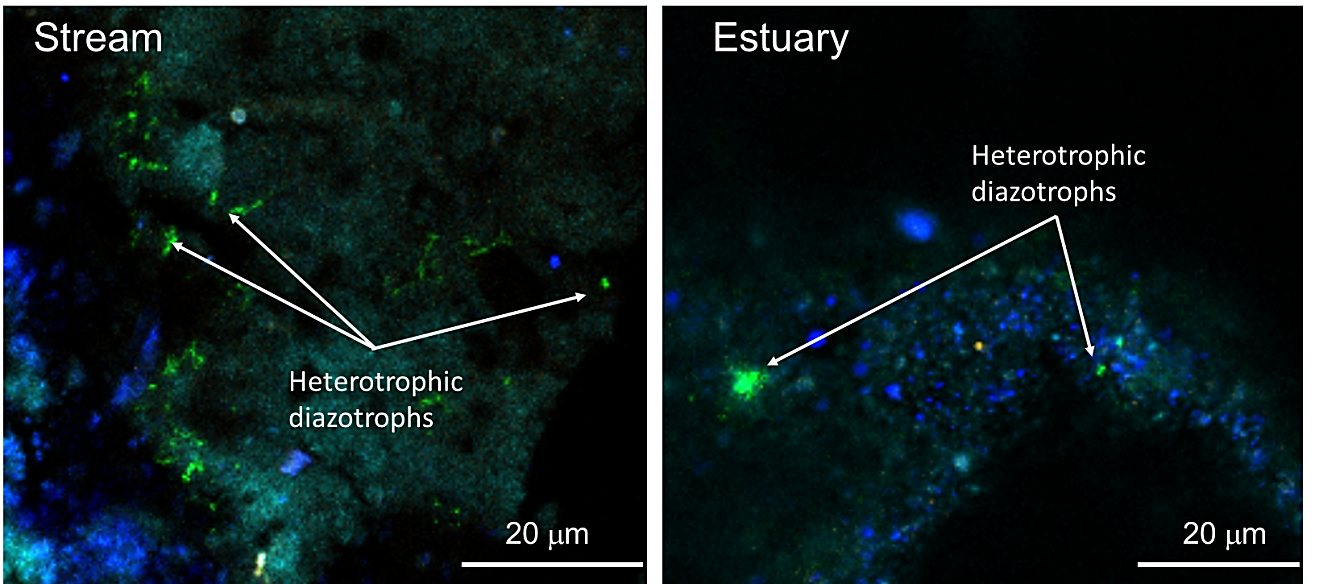


**Figure S5** - Magnified images of heterotrophic diazotrophs (green) that have synthesized the nitrogenase enzyme and captured associated with aggregate comprise a polysaccharide matrix (stained by Con A, turquoise). These aggregates also comprised other microorganisms (stained by DAPI, blue) and cyanobacterial cells (identified by the auto florescence of phycoerythrin
